# Anti-Inflammatory Metabolites in the Pathogenesis of Bacterial Infection

**DOI:** 10.3389/fcimb.2022.925746

**Published:** 2022-06-15

**Authors:** Andreacarola Urso, Alice Prince

**Affiliations:** Department of Pediatrics and Pharmacology, Vagelos College of Physicians & Surgeons Columbia University, New York, NY, United States

**Keywords:** adenosine, itaconate, metabolism, anti-inflammatory, bacterial infections, infection tolerance

## Abstract

Host and pathogen metabolism have a major impact on the outcome of infection. The microenvironment consisting of immune and stromal cells drives bacterial proliferation and adaptation, while also shaping the activity of the immune system. The abundant metabolites itaconate and adenosine are classified as anti-inflammatory, as they help to contain the local damage associated with inflammation, oxidants and proteases. A growing literature details the many roles of these immunometabolites in the pathogenesis of infection and their diverse functions in specific tissues. Some bacteria, notably *P. aeruginosa*, actively metabolize these compounds, others, such as *S. aureus* respond by altering their own metabolic programs selecting for optimal fitness. For most of the model systems studied to date, these immunometabolites promote a milieu of tolerance, limiting local immune clearance mechanisms, along with promoting bacterial adaptation. The generation of metabolites such as adenosine and itaconate can be host protective. In the setting of acute inflammation, these compounds also represent potential therapeutic targets to prevent infection.

## Introduction

Bacterial infections, especially those due to antimicrobial- resistant organisms, are a worldwide public health problem (Murray et al., 2022). While resistance has imposed treatment challenges, substantial mortality is nonetheless associated with pathogens that are entirely susceptible to available therapies. This suggests the influence of other factors enabling successful infection. The importance of host-and-pathogen-derived metabolites, which impact bacterial adaptation and shape the nature of the immune response, is increasingly recognized. Proinflammatory immunometabolites are critical in activating host defenses, and their anti-inflammatory counterparts function to prevent associated toxicities. As a consequence of effects on host immunity and bacterial survival, the fluctuation of pro and anti-inflammatory metabolites has profound effects in determining the outcome of infection.

The innate immune response to bacteria is initiated by ligand-receptor interactions to bacterial components or pathogen associated molecular patterns (PAMPS) such as lipopolysaccharide (LPS). Pattern recognition receptors (PRRs) on macrophages, such as TLR4, activate the transcription of NF-κB to initiate expression of proinflammatory cytokines and chemokines. Along with protein secretion, macrophages undergo metabolic reprogramming, shifting from their resting state of oxidative phosphorylation to use aerobic glycolysis (Peace and O’Neill, 2022). This process leads to the succinate-mediated activation of the transcription factor HIF1α and the production of IL-1β, which favor the differentiation of CD4^+^ Th17 and Th1 effector cells (Peace and O’Neill, 2022). Equally important are the proteins and metabolites that suppress inflammation and act to prevent the ensuing damage caused by neutrophil oxidants and proteases. A component of this regulatory process is HIF-1α, a transcription factor that drives the expression of *acod1* or *irg1* generating the anti-inflammatory product itaconate (Peace and O’Neill, 2022). The recruitment of regulatory lymphocytes also contributes by dampening inflammation *via* the ectonucleotidase-mediated synthesis of adenosine, a potent anti-inflammatory molecule. These metabolites, which we will discuss in detail, function to prevent tissue damage. However, metabolites countering the immune response to infection can have major roles in promoting bacterial persistence.

Upon infection, pathogens must rapidly adapt their metabolism to compete for and efficiently utilize available substrates. The release of anti-inflammatory metabolites at the site of infection can affect pathogenesis in two major ways: It can alter the function of host immune cells and it can drive changes in bacterial metabolic activity, selecting for the variants that are optimally fit to persist. This creates a setting of tissue tolerance, in which the host-adapted pathogen and the locally immunosuppressed host generate a state of persistent infection (Schneider and Ayres, 2008; Wong Fok Lung et al., 2022), as found in TB, COPD, cystic fibrosis and other common infections.

In this review we highlight two major immunometabolites, adenosine and itaconate, both of which promote bacterial metabolic responses and inactivate host immune effectors. We aim to highlight the substantial effects of these abundant metabolites on immune clearance mechanisms, reviewing the biochemical and immunological alteration of host defenses. We will also examine how different bacterial species respond to adenosine and itaconate, depending upon their ability to metabolize each potential substrate or exploit its immune effects.

## Adenosine Mediates Pro and Anti-Inflammatory Cascades

### Synthesis and Biology of Adenosine

Adenosine belongs to a class of molecules known as purines, which are heterocyclic aromatic compounds including nucleotides (adenine and guanine), deoxynucleotides (deoxyadenine and deoxyguanine) and ribonucleotides (adenosine and guanosine) required for the cellular processes of DNA and RNA replication. Adenosine can be synthesized intracellularly and extracellularly. Intracellular adenosine is generated through S-adenosylhomocysteine hydrolase (SAHases). S-adenosylhomocysteine is converted to adenosine which is secreted to the extracellular space mainly through equilibrative nucleoside transporters (ENT1-4) and concentrative nucleoside transporters (CNT1-3) (Dal Ben et al., 2018).

**Figure 1:**
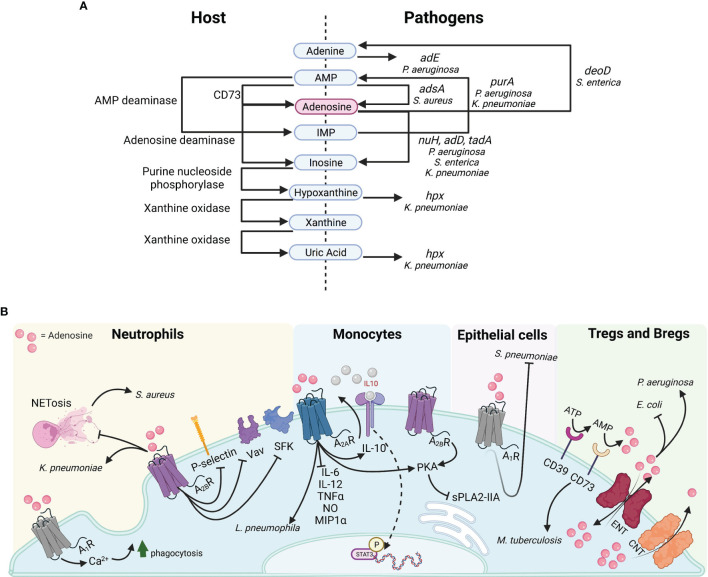
Purine metabolism and adenosine signaling in bacterial pathogenesis. **(A)** Host proteins (left) and pathogen genes (right) illustrating the synthesis or catabolism of purinergic compounds. **(B)** Adenosine targets diverse receptors on multiple cell types.

Extracellular adenosine is mainly synthesized through the ectonucleotidases CD39 and CD73 (**Figures 1A, B**). In the setting of infection, pro-inflammatory adenosine triphosphate (ATP) is dephosphorylated to adenosine monophosphate (AMP) *via* the ecto-nucleotide triphosphate hydrolase-1, or CD39, in a Ca^2+^ and Mg^2+^ dependent manner (Kaczmarek et al., 1996). The AMP product is then rapidly converted to adenosine *via* the ecto-5’-nucleotidase, or CD73 (Antonioli et al., 2013). Therefore, the anti-inflammatory role of adenosine is partly attributed to the reduction of extracellular ATP required for its synthesis (Silva-Vilches et al., 2018). CD73 and CD39 deficient mice are common mouse models in studies of the purinergic system as they show substantial decrease in adenosine levels. A non-canonical synthesis pathway involves the hydrolysis of extracellular nicotinamide adenine nucleotide (NAD+) to generate adenosine diphosphate ribose (ADPR) *via* the enzyme CD38. ADPR is next hydrolyzed to AMP by CD203a, after which CD73 mediates adenosine conversion (Ferretti et al., 2019).

Intracellular regulation of adenosine synthesis occurs through purinergic negative feedback loops. However, once secreted, extracellular concentration is regulated by either its conversion to inosine, xanthine and uric acid *via* adenosine deaminases (Ada*)* or xanthine oxidases (Xo), or by reuptake (Ferretti et al., 2019). Of note, both Gram positive bacteria such as *Staphylococcus aureus*, and Gram negatives like *Pseudomonas aeruginosa, Klebsiella pneumoniae*, and *Salmonella enterica* produce enzymes facilitating purine degradation for nitrogen and carbon scavenging, as well as adenosine synthesis for immune evasion (**Figure 1A**).

### Pro and Anti-Inflammatory Effects of Adenosine Signaling

Adenosinergic signaling occurs *via* four subtypes of G-protein coupled receptors A_1_, A_2A_, A_2B_, A_3_, which are all coupled to mitogen activated protein kinase (MAPK) pathways such as ERK1, ERK2, c-JUN N-terminal kinase, and p38 MAPK (Antonioli et al., 2013). Despite their affinity for the same ligand, the four adenosine receptors are paired with distinct second messenger pathways, which promote a diverse array of inflammatory and anti-inflammatory phenotypes across tissues. The abundance and selectivity of these receptors are a major factor in mediating the downstream consequences of adenosine signaling (**Figure 1B**). The low affinity receptors A_1_ and A_3_ can be coupled to G_i_, G_o_ or G_q_ proteins, resulting in decreased cyclic adenosine monophosphate (cAMP) and increased calcium ions. The A_1_ receptor is associated with both anti-inflammatory and proinflammatory effects. In the peripheral nervous system, adenosine binding to the A_1_ receptor can elicit analgesic effects in neuroinflammation. Conversely, in innate immune cells and solid organs chronic adenosine exposure mediates proinflammatory effects upon A_1_ receptor binding (i.e. bronchoconstriction in the lung; negative chronotropic effects in the heart atria; reduced insulin secretion in the pancreas; and reduced blood flow and renin release in the kidneys) (Borea et al., 2018; Le et al., 2019). The A_3_ receptor largely mediates anti-inflammatory effects in mucosal sites and immune cells (Borea et al., 2018). Analogously, the A_2A_ and A_2B_ receptors, which are high affinity receptors can be coupled to either G_s_ or G_q,_ resulting in increased cAMP levels. These serve primarily the role of inflammatory suppressors (Borea et al., 2018). These receptors are highly concentrated on innate and adaptive immune cells for their regulation. Among the two, only the A_2B_ subtype has been linked to some pro-inflammatory outcomes. In the intestine and lung, adenosine binding to the A_2B_ receptor contributed to inflammation in hypoxia. In microglia, A_2B_ binding increased IL-6 production and hypersensitive nociception (Hu et al., 2016; Merighi et al., 2017; Bowser et al., 2018; Le et al., 2019).

### Adenosine Promotes Anti-Inflammatory Phenotypes in Myeloid Cells

Adenosine has potent immunoregulatory effects on inflamed tissues. Upon infection or injury, adenosine is synthesized to prevent excessive damage caused by effector cells of innate and adaptive origin. In monocytes, adenosine reduces inflammation through the activation of A_2A_, A_2B_, A_3_ receptors by modulating cytokine secretion, DNA binding and intracellular signaling pathways (**Figure 1B**). Adenosine restricts the secretion of pro-inflammatory cytokines IL-6, IL-12, TNFα, NO and MIP1α from macrophages and specifically promotes the conversion of M1 monocytes to the anti-inflammatory M2 phenotype (Ferrante et al., 2013). Intracellularly, it interferes with Akt signaling in monocytes by inhibiting IκB-α degradation, thus preventing NF-κB DNA binding and stimulating the anti-inflammatory IL-10-induced STAT3 signaling (Lee et al., 2011; Koscsó et al., 2013). High concentrations of adenosine occur in response to active inflammatory processes, which demand continuous supply of anti-inflammatory agents for their regulation. Indeed, the cAMP/CREB second messenger pathway, activated upon A_2A_ and A_2B_ binding, transcriptionally regulates CD39 expression, suggesting a positive feedback loop for adenosine synthesis. The increased adenosine concentration prolongs TLR inhibition and solidifies the M2 phenotype characterized by a decrease in glycolytic rate. In the context of bacterial infection, high adenosine concentrations inhibit macrophage phagocytosis, thus promoting colonization (Frasson et al., 2017).

Purinergic signaling affects neutrophils by both promoting their antibacterial functions, as well as by attenuating chemotaxis and adhesion (**Figure 1B**). Specifically, stimulation of the A_1_ receptor on neutrophils results in increased phagocytosis and reactive oxygen species production which contributes to their proinflammatory nature. In contrast, both adenosine and A_2B_ receptor agonists inhibit P-selectin/β2 integrin-mediated neutrophil rolling, as well as the activation of SFKs and Vav guanine-nucleotide exchange proteins, which mediate neutrophil cytoskeletal rearrangement (Yago et al., 2015). These effects on neutrophil function limit the intensity of proinflammatory responses to injury and pathogens. Indeed, phagocytosis and ROS secretion are reduced upon A_2B_ activation in neutrophils responding to bacterial infection (Frasson et al., 2017).

### Adenosine Contributes to Bacterial Pathogenesis

The impact of adenosine signaling in the pathogenesis of infection has been studied with a variety of pathogens and model systems yielding differing results. While adenosine is important to prevent tissue damage, it can contribute to bacterial pathogenesis by disrupting immune defenses. Inflammation in LPS-challenged tissues, such as the lung and vasculature, stimulates the protective release of adenosine to halt the damage induced by monocytes and granulocytes (Gonzales et al., 2014; Kutryb-Zajac et al., 2019). In this setting, pathogens can exploit the anti-inflammatory effects of adenosine on the innate and adaptive immune response (**Figure 1B**).

In a model of *Myobacterium tuberculosis* infection in CD73 deficient mice, which are severely restricted in their extracellular adenosine synthesis, bacterial clearance is enhanced (Petit-Jentreau et al., 2015). Of note, this effect was not dependent upon macrophage CD73 activity, as *in vitro* incubation with ATP and bacteria did not affect their function. Instead, in these mice *M. tuberculosis* infection led to increased TNFα, IL-6 and KC levels along with higher numbers of polymorphonuclear neutrophils in mouse lungs, which enhance clearance (Petit-Jentreau et al., 2015). These results illustrate the complexity of the adenosine-receptor interaction depending upon the specific immune cells involved in host defense.

In the pathogenesis of *K. pneumoniae* respiratory infection, adenosine binds to A_2B_ receptors on neutrophils inhibiting phagocytic clearance and decreasing killing (Barletta et al., 2012). By disrupting the ligand-receptor interaction, A_2B_ deficient mice were found to have improved clearance of *K. pneumoniae* compared to WT mice. Additional studies indicate that in WT mice adenosine binding prevents extracellular DNA accumulation and neutrophil extracellular trap (NET) formation. These NETs are outgrowths of neutrophils, containing histones and DNA which contribute to bacterial capture and clearance (Barletta et al., 2012). The reduced NETosis mediated by adenosine signaling in the WT host benefits *K. pneumoniae* survival.

The Gram positive bacterium *Staphylococcus aureus* exploits adenosine accumulation through several mechanisms. *S. aureus* expresses a surface protein adenosine synthase A (AdsA) that generates adenosine from ATP, ADP and deoxyadenosine, as well as the cytotoxic deoxyguanosine (Winstel et al., 2018; Tantawy et al., 2022). In a model of renal abscess and septicemia, the resulting NETosis provides *S. aureus* with a source of DNA and nucleosides for adenosine synthesis. The released adenosine then induces caspase-3-dependent macrophage apoptosis (Thammavongsa et al., 2009; Thammavongsa et al., 2011), furthering *S. aureus* proliferation. An additional response promoting *S. aureus* survival is blocking the activation of type IIA secretory phospholipase A2 (sPLA2-IIA) (Pernet et al., 2015). *S. aureus* generation of adenosine *via* AdsA enables the organism to escape sPLA2-IIA-mediated killing by impairing macrophage phagocytosis. In these model systems, *S. aureus* successfully escapes innate immune responses by contributing to the adenosine pool and preventing macrophage killing (Winstel et al., 2018). AdsA-generated adenosine also restrains protective Th1 and Th17 responses demonstrated in a model of intraperitoneal infection. In this situation adenosine reduces caspase-1-mediated NLRP3 inflammasome activation and IL-1β secretion (Deng et al., 2021).

Besides phagocytes, other classes of immune cells participate in adenosine – mediated immune function in infection. The specialized adaptive sub-lineages of Regulatory T and B lymphocytes, which possess CD39/CD73 ectonucleotidase complexes, suppress effector cells by generating adenosine (Antonioli et al., 2013). Sepsis-survival models of *Legionella pneumophila* infection exhibited increased CD39-expressing B cells, elevated extracellular adenosine and impaired bacterial killing (Nascimento et al., 2021). In this model, adenosine-mediated inhibition of splenic macrophages relied on both CD39, for adenosine synthesis, and A_2A_ for adenosine binding. In CD39 deficient (Ent-/-) mice or with the blockade or deletion of A_2A_ there was both reduced bacterial burden and enhanced host resistance to *L. pneumophila* in spleen and lung (Nascimento et al., 2021).

*In vivo* studies of adenosine and ATP in the response to infection reflect considerable variability depending upon both the pathogen and the infected tissue. In the lung, injections of adenosine or ATP prior to or 3-hours-post-intratracheal inoculation with *Escherichia coli* protected the host from infection and stimulated proinflammatory recruitment (Gross et al., 2020). These protective effects are similar to the phenotype observed with *Streptococcus pneumoniae* infections, in which adenosine and ATP through the A_1_ receptor prevent pathogen adhesion to pulmonary epithelial cells (Bhalla et al., 2020). ATP release is a damage associated molecular pattern (DAMP), a signal of host damage to which the innate immune system promptly responds. However, in a systemic model of *E. coli infection*, LPS-induced ATP release served as substrate for adenosine synthesis, resulting in diminished proinflammatory recruitment and successful establishment of infection (Kondo et al., 2019). These results suggest that the anti-inflammatory effects of adenosine may be deleterious for the host, and enable bacterial infection in specific tissues.

### Pathogens Catabolize Adenosine and Its Derivatives

One of the major explanations for the varied responses to adenosine in different bacterial infections is the ability of some pathogens to utilize it as a carbon and nitrogen source (Matsumoto et al., 1978). These metabolic degradative pathways are differentially expressed in specific organisms and their impact is not appreciated in studies using LPS as a surrogate for Gram negative bacteria. As a suitable nitrogen reservoir, adenine is generated through a network of purinergic enzymes in *P. aeruginosa*, which possesses enzymes for both inosine and adenosine monophosphate (IMP, AMP) synthesis (*purA-D*), as well as adenosine deamination and adenosine and inosine degradation (*nuh*, *add*) (**Figure 1A**) (Heurlier et al., 2005; Goble et al., 2011). In *P. aeruginosa*, the major quorum sensing regulator LasR modulates genes *nuh* and *add*, which are determinants of successful growth on adenosine and inosine (Heurlier et al., 2005). Thus, beyond its immunomodulatory effects, excess adenosine at infected host sites can promote pathogen growth. Indeed, profiling of *P. aeruginosa* clinical strains indicated that biofilm-forming isolates preferred amino acids such as L-threonine and L-serine, while non-biofilm-forming isolates utilized adenosine and inosine to proliferate (Ismail et al., 2020).

*K. pneumonia* readily metabolizes purines and utilizes the adenosine product hypoxanthine as a nitrogen source (de la Riva et al., 2008). To initiate this process, the phosphorylated nitrogen regulator NtrC-P binds to an enhancer activating the *hpx* gene cluster, associated with oxidation of nitrogenous compounds, specifically hypoxanthine and uric acid (**Figure 1A**) (de la Riva et al., 2008). During infection, one of the greatest stressors for pathogens is scarcity of preferred nutrients. The *hpx*DE operon system is activated in response to nitrogen limitation and to the presence of available purinergic compounds (de la Riva et al., 2008). The evolution of NMT1 motif xanthine riboswitches and adenosine deaminases (*tada*) to prevent purine toxicity is a known adaptive function of *K. pneumoniae* (Guzmán et al., 2011; Yu and Breaker, 2020). Exposure to adenosine precursors such as IMP enhances its hypermucoviscosity and production of capsular polysaccharide (CPS), both associated with infection of the alveolar epithelium (Mike et al., 2021). The ability to hydrolyze and synthesize purines has been associated with the intracellular persistence of both classical and hypervirulent strains of *K. pneumoniae* in lung infection (Bachman et al., 2015; Bruchmann et al., 2021). Mutations in the cytoplasmic protein adenylosuccinate synthase (*purA*) prevented *K. pneumoniae* from repurposing intracellular nucleosides for CPS biosynthesis and exhibited growth defects along with mutants *purF*, *purL* and *purH* (Mike et al., 2021). The release of ATP and its function as a host innate immune signal also fuels the adaptation of these common pathogens to the site of infection.

While adenosine catabolism is a shared property of several bacterial species, its metabolic cost is still poorly understood. *In vitro* and *in vivo* studies of *Salmonella enterica* in intestinal epithelial cells describe reduced bacterial colonization upon adenosine exposure due to inhibition of pathogen growth. *S. enterica* express the adenosine-converting enzymes adenosine deaminase (*add*) and purine nucleoside phosphorylase (*deoD*), which convert adenosine to inosine and adenine, respectively (**Figure 1A**) (Kao et al., 2017). While *S. enterica* enzymatic activity promoted bacteriostatic conditions when incubated with adenosine *in vitro*, *deoD* and *add* deficient strains were able to reach exponential growth (Kao et al., 2017). The selective pressure exerted by adenosine on *S. enterica* discourages its consumption and suppresses virulence. Similarly, in CD73 deficient mice lacking adenosine accumulation, there is increased transepithelial migration of the pathogen compared to WT mice, suggesting that adenosine has important protective effects on the host to the detriment of *S. enterica* (Kao et al., 2017).

## Itaconate Participates in Anti-Inflammatory Cascades

### Itaconate Synthesis and Biology

**Figure 2:**
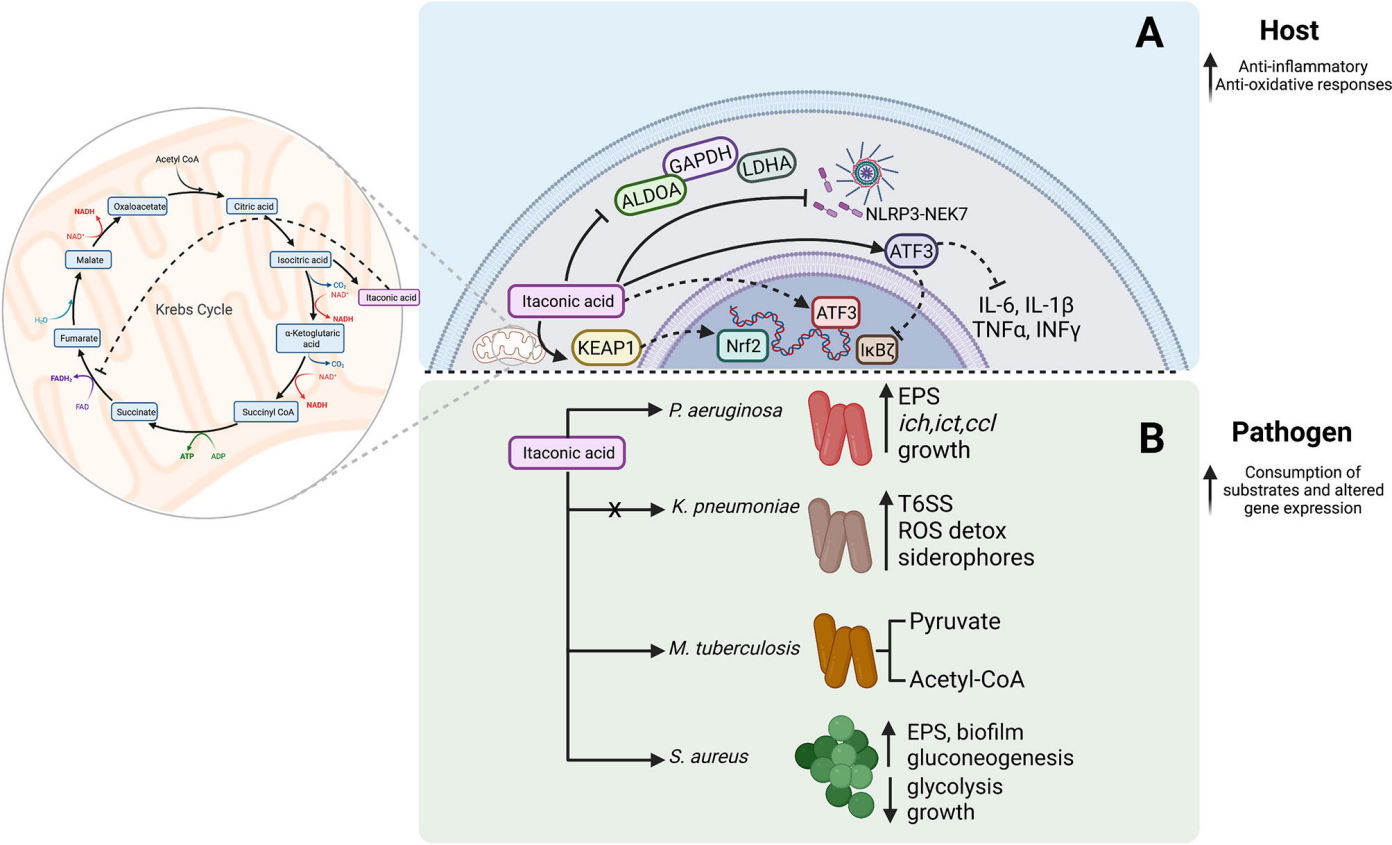
Itaconate effects on host signaling and bacterial survival. **(A)** Itaconate targets host signaling and suppresses inflammation. **(B)** Itaconate fuels bacterial metabolism and adaptation.

Itaconate is among the most abundant metabolites produced by macrophages. It is a TCA metabolite derived from the conversion of an intermediate of cis-aconitate by cis-aconitate decarboxylase (CAD), also known as aconitate decarboxylase 1 (ACOD1) or immunoregulatory gene 1 (IRG1) (Michelucci et al., 2013). Itaconate interrupts the TCA cycle in mitochondria at the enzymatic level of succinate dehydrogenase (SDH) (**Figure 2**) (Cordes et al., 2016; Lampropoulou et al., 2016). The inhibition of complex-II oxidation is mediated by the structural similarity between itaconate and succinate, where itaconate accumulation is translated to a negative feedback signal (Lampropoulou et al., 2016). Upon loss of *irg1*, myeloid reliance on respiration in cell culture is restored potentially through anaplerosis and a functional succinate-Q oxidoreductase. In the context of infection, itaconate reduces the TLR-triggered secretion of inflammatory cytokines (Li et al., 2013). Specifically, in mouse models of pneumonia, itaconate was identified as a common molecule in the airway metabolome contributing to bacterial pathogenesis (Riquelme et al., 2020; Tomlinson et al., 2021; Wong Fok Lung et al., 2022).

### Anti-Inflammatory Effects of Itaconate Signaling

The biochemical properties of itaconate contribute to the anti-inflammatory profile of macrophages (**Figure 2A**) (Strelko et al., 2011). The use of proteomic screens indicated that itaconate-mediated chemical alteration of cytosolic targets KEAP1, ATF3, NF-κB, and cysteine modifications in NLRP3 and glycolytic enzymes could be responsible for the immune effects that were observed. The unsaturated dicarboxylic acid structure of itaconate renders it slightly electrophilic and mediates an interaction with thiol functional groups through 2, 3-dicarboxypropylation in the cytosol (Hooftman et al., 2020; Peace and O’Neill, 2022). Itaconate has anti-inflammatory and anti-oxidant properties that have been partly ascribed to itaconate-induced KEAP1 protein alkylation. This modification boosts Nrf2 and glutathione levels in myeloid cells promoting an anti-inflammatory phenotype (Mills et al., 2018). ATF3 is also targeted by itaconate, altering the inflammatory profile of macrophages by inhibiting proinflammatory cytokine release (IL-6, IL-1β, TNFα, INFγ). ATF3 can be upregulated by exogenous and endogenous itaconate, which interferes with the NF-κB inhibitor zeta (IκBζ) axis thus reducing pro-inflammatory cytokine secretion (Bambouskova et al., 2018). This phenotype reversed in the *atf3*-knockout or *irg1*-knockout cell line which remained proinflammatory (Bambouskova et al., 2018).

Itaconate is also involved in amino acid modifications (**Figure 2A**). The itaconate-induced Cys548 modification interferes with the NLRP3-NEK7 interaction, inhibiting inflammasome activation and IL-1β secretion in macrophages, thus promoting an anti-inflammatory phenotype (Hooftman et al., 2020). Additional targets for cysteine modifications include key glycolytic enzymes such as GAPDH, aldolase (ALDOA) and lactate dehydrogenase A (LDHA), of which ALDOA holds the most upstream position converting β-D-fructose-1,6-phosphate to D-glyceraldehyde-3-phosphate and dihydroxyacetone phosphate (Qin et al., 2019). Among the targets Cys339 was found to be a functional residue leading to the instability of the protein after the itaconate modification. Overall, a substantial body of evidence in diverse systems confirms a role for itaconate as a major metabolic regulator of glycolysis and as such could be important in host immune responses and disease tolerance (Domínguez-Andrés et al., 2019; Qin et al., 2019)

### Itaconate Contributes to Bacterial Pathogenesis

Itaconate is a host-generated metabolite, initially thought to function as an antimicrobial agent due to its effects on isocitrate lyase-mediated glyoxylate shunt inhibition (McFadden et al., 1971; McFadden and Purohit, 1977; Nair et al., 2018). However, opportunistic pathogens exhibit diverse metabolic and transcriptional alterations in response to increased itaconate levels which extend beyond mammalian immunity. Accumulating evidence indicates that itaconate has major effects on both bacterial metabolic activity as well as the host immune function. For example, in specific disease settings, such as cystic fibrosis, limited activity of the phosphatase and tensin (PTEN) protein results in the accumulation of both succinate and itaconate in the airway which have profound effects on bacterial metabolism as well as on the host inflammatory response to infection (Riquelme et al., 2017).

Just as many Gram negative bacteria are able to utilize adenosine released by the host, *P. aeruginosa* clinical isolates catabolize itaconate *via* three devoted genes (*ict*, *ich*, and *ccl)* expressed to use itaconate as a major carbon source (**Figure 2B**) (Riquelme et al., 2020). In comparison to the laboratory strain PAO1, growth of *P. aeruginosa* clinical isolates is significantly boosted in *irg1-*competent mice compared to *Irg1^-/-^*, where adapted strains exhibit increased proficiency in establishing infection (Riquelme et al., 2020). In addition to using itaconate as a carbon source, this dicarboxylate also drives major adaptive changes *in P. aeruginosa* metabolism. Exposure to itaconate results in increased utilization of the Entner-Doudroff pathway and the glyoxylate shunt, fueling pathways that lead to increased production of extracellular polysaccharides (EPS) and decreased display of LPS (Riquelme et al., 2019; Riquelme et al., 2020). Such host adapted strains respond to itaconate with the upregulation of EPS genes such as *algT*, *algA*, *algD*, *algQ* and *mucA* (Riquelme et al., 2020) which promote biofilm formation. This lifestyle provides defense against antibiotics, antimicrobial peptides, oxidants and phagocytosis enhancing bacterial persistence. Furthermore, EPS itself stimulates myeloid cells to release additional itaconate, which is then exploited by *P. aeruginosa* as a carbon source (Riquelme et al., 2020).

Itaconate metabolism is also an important factor holding a multidimensional role in the success of the airway pathogens *Aspergillus terreus* and *Myobacterium tuberculosis* (Bonnarme et al., 1995; Wang et al., 2019). Itaconate was first identified as an inhibitor of methylmelonyl-CoA mutase *in vitro* preventing *M. tuberculosis* growth on permissive media (Ruetz et al., 2019). However, *M. tuberculosis* can also generate itaconyl-CoA, which is hydrated to form (S)-citramalyl-CoA and lysed into pyruvate and acetyl-CoA through Rv2498c, a stereospecific bifunctional β-hydroxyacyl-CoA lyase (**Figure 2B**). Through this common mechanism, *M. tuberculosis* and *A. terreus* are able to resist growth inhibition and itaconate toxicity, and proliferate by utilizing the generated byproducts (Sasikaran et al., 2014; Chen et al., 2016; Wang et al., 2019). Thus, itaconate, like adenosine, may promote infection by supporting bacterial proliferation and by suppressing immune activation.

Even organisms that do not metabolize itaconate can benefit from its presence by altering their own metabolic activity to thwart immune clearance. The expression of *Irg1* by myeloid cells is also a major component of the anti-inflammatory milieu leading to infection tolerance (Wong Fok Lung et al., 2022). Metabolically active *K. pneumoniae* ST258 strains induce ROS-generating pathways, myeloid-derived suppressor cell (MDSCs) recruitment, and abundant itaconate release in the airway (Ahn et al., 2016; Wong Fok Lung et al., 2022). Itaconate helps to control *K. pneumoniae* infection, as there is a significantly increased bacterial burden in *Irg1* ^-/–^mice (Wong Fok Lung et al., 2022). However, the presence of itaconate enables the infected mice to tolerate remarkably high levels of infection (Wong Fok Lung et al., 2022). Bulk RNA-sequencing of *K. pneumoniae* infected *Irg1* ^-/–^lung reflects how itaconate creates a milieu that enables infection tolerance. In the *Irg1*-/- mice ST258 *K. pneumoniae* organisms increase the expression of glutathione-mediated ROS detoxification (peroxidases, S-transferases, *gsiD*, *soxR*, *aphA*), siderophore production (*entS, fepA/D/G, fes*), and type six secretion system (T6SS) gene transcription, reflecting the excess oxidant stress that is normally controlled by itaconate (**Figure 2B**) (Wong Fok Lung et al., 2022).

The Gram positive *S. aureus* cannot utilize itaconate as a carbon source. Exposure to itaconate imposes metabolic stress by suppressing aldolase activity and interfering with glycolysis, the preferred metabolic pathway in *S. aureus* (Tomlinson et al., 2021). Gluconeogenesis is upregulated in response to itaconate exposure which promotes the selection of strains shunting carbohydrates in EPS and biofilm (**Figure 2**, Pathogen) (Tomlinson et al., 2021). Thus, itaconate promotes a metabolic phenotype in *S. aureus* favoring persistent infection.

## Therapeutic Targeting of Immunometabolites in Bacterial Infection

We have briefly highlighted some of the major consequences of two abundant immunometabolites, adenosine and itaconate, in the pathogenesis of bacterial infection (**Figures 1** and **2**). We illustrate how anti-inflammatory metabolites may have both beneficial and negative consequences on host health. Suppressing inflammation through both itaconate and adenosine is permissive of neoplastic diseases (Weiss et al., 2018; Churov and Zhulai, 2021). We learn from oncology that the efforts to interfere with tumor metabolism can be therapeutic and strategies modulating immune cell metabolic activity are being pursued (Stine et al., 2022).

Therapeutic approaches independently targeting host or bacterial gene products have been largely unsuccessful likely due to bacterial metabolic adaptation to the selective pressures imposed during infection (Opoku-Temeng et al., 2019; Wang et al., 2021; Jahantigh et al., 2022). It is increasingly evident that upon infection, metabolically active bacteria rapidly alter gene expression and survival strategies (Wong Fok Lung et al., 2022). To prevent bacterial persistence, we could similarly identify conserved metabolic targets, such as the anti-oxidant and protective glyoxylate shunt, or catabolic targets, permitting substrate consumption. Anti-inflammatory metabolites and existing pharmacological agents could be combined to mitigate host damage and reduce bacterial colonization, as recently indicated in *S. aureus* bacterial pneumonia and endopthalamitis (Liu et al., 2021; Singh et al., 2021). A similar strategy has been shown in an *in vitro* model of *P. aeruginosa* treated with a combination of itaconic acid and tobramycin to penetrate biofilm (Ho et al., 2020). In this era of precision medicine, it should be possible to identify the antimicrobial susceptibility of infecting organisms along with critical metabolic pathways mediating their survival *in vivo*. As a step towards improving our understanding of persistent bacterial infection, it is necessary to simultaneously investigate both host and pathogen in their metabolic interactions, and how they shape the immune response and bacterial metabolic adaptation.

## Author Contributions

AU and AP conceived the project and wrote the manuscript. All authors contributed to the article and approved the submitted version.

## Funding

NSF (AU) - DGE 2036197. NIH (ASP) - 5R35HL135800-06.

## Conflict of Interest

The authors declare that the research was conducted in the absence of any commercial or financial relationships that could be construed as a potential conflict of interest.

## Publisher’s Note

All claims expressed in this article are solely those of the authors and do not necessarily represent those of their affiliated organizations, or those of the publisher, the editors and the reviewers. Any product that may be evaluated in this article, or claim that may be made by its manufacturer, is not guaranteed or endorsed by the publisher.
